# Fibroblast Growth Factor 11 Enables Tumor Cell Immune Escape by Promoting T Cell Exhaustion and Predicts Poor Prognosis in Patients with Lung Adenocarcinoma

**DOI:** 10.1155/2023/9303632

**Published:** 2023-05-19

**Authors:** Baoqian Zhai, Jiacheng Wu, Tao Li

**Affiliations:** ^1^Department of Radiotherapy Oncology, Yancheng City No. 1 People's Hospital, The Fourth Affiliated Hospital of Nantong University, Yancheng 224005, China; ^2^Department of Urology, Affiliated Tumor Hospital of Nantong University & Nantong Tumor Hospital, No. 30, Tongyang bei Road, Tongzhou District, Nantong 226361, China; ^3^Department of Medical Oncology, Affiliated Tumor Hospital of Nantong University &Nantong Tumor Hospital, No. 30, Tongyang bei Road, Tongzhou District, Nantong 226361, China

## Abstract

Fibroblast growth factor 11 (FGF11) accelerates tumor proliferation in a variety of cancer types. This study aimed to examine the link between FGF11 and the prognosis of lung adenocarcinoma. FGF11 was searched in the Tumor Cancer Genome Atlas (TCGA) and ImmProt databases. The link between FGF11 and lung cancer clinical data was investigated using TCGA and Kaplan–Meier (KM)-plotter databases, and we developed a prediction model. Putative mechanisms of action were investigated using Gene Ontology (GO) and KEGG enrichment analyses. The GeneMANIA and STRING databases were used to search for genes that interact with FGF11, and the Tumor Immune Estimation Resource (TIMER) database was used to discover connections between FGF11 and immune cells, as well as any correlations with immune-related genes. We found that FGF11 expression was higher in the lung adenocarcinoma tissue than in the paracancerous tissue, and patients with high FGF11 expression had a lower overall survival, progression-free survival, and disease specific survival rate than those with low FGF11 expression. The expression of FGF11 was inversely linked to six types of infiltrating immune cells in the TIMER database and was associated with EGFR, VEGFA, BRAF, and MET expressions. The FGF11 gene is negatively correlated with the expression of most immune cells, mainly with various functional T cells including Th1, Th1-like, Treg, and Resting Treg characterization genes. These results indicate that FGF11 has the potential to be a new lung adenocarcinoma biomarker. It increases tumor cell immune escape by boosting T cell exhaustion in the tumor microenvironment, contributing to the poor prognosis of the patients with lung adenocarcinoma. These results provide incentive to further research FGF11 as a possible biomarker and drug target for patients with lung adenocarcinoma.

## 1. Introduction

Lung cancer remains prevalent and is a leading cause of cancer-related mortality globally, accounting for 18.4 percent of all cancer-related deaths [1, 2]. Lung adenocarcinoma is a prominent type that accounts for more than half of all lung cancer cases [3]. Lung cancer begins in the bronchial epithelium and mucous glands of the major bronchi. Although it has a lower incidence than squamous cell carcinoma or undifferentiated carcinoma, it typically develops at a younger age. Small bronchial adenocarcinomas are the most common type of adenocarcinoma and manifest as peripheral lung cancer. In the early stages, there are usually no noticeable symptoms, and they are often discovered during chest radiography. On imaging, the tumor appears as a slow-developing round or oval mass. Although hematogenous metastasis may develop throughout the progression of cancer, lymphatic metastasis is more common later in cancer development [4, 5]. Lung adenocarcinoma has a 5-year survival rate of approximately 20.0%–30.0% [6, 7]. Previous treatment approaches, such as minimally invasive surgery, radiation, and chemotherapy, have progressively improved, and the survival time of lung adenocarcinoma patients has accordingly increased [8]. The risk of postoperative recurrence and death in patients with early-stage lung adenocarcinoma can be reduced by immediate surgery [9]. Therefore, the identification of reliable target molecules for early detection and therapy is critical.

Fibroblast growth factors (FGFs), particularly FGF1-23, have been identified in humans and rats in the previous studies [10]. FGFs are classified as either secreted (FGF1 and FGF10) or intracellular (FGF11 and FGF14) [11]. Most secreted FGFs and their external FGF receptors are widely studied in malignancies, and they play well-defined biological functions. FGF10/FGFR2 signaling promotes pancreatic cancer by increasing cell motility and invasion [12]. FGF15 inhibits bile acid metabolism and cancer by activating Hippo signaling [13]. FGF20, produced by glioma cells, activates *β*-catenin, which limits the anticancer macrophage activity [14]. The role of intracellular nonsecreted FGFs, also known as FGF homologous issues (FGF11-FGF14), is unknown. According to a previous study, hypoxia increases the thyroid cancer growth via the HIF1/FGF11 feedback loop [15]. By modulating FGF11, miRNA-541, androgen receptor (AR), and MMP9 signaling, infiltrating T cells increase prostate cancer metastasis [16].

By combining the Cancer Genome Atlas (TCGA) database analysis with immune infiltration-related information, we were able to identify the differentially expressed gene FGF11, which has been linked to lung adenocarcinoma (LUAD) prognosis. In this study, we explore the effect of FGF11 expression on tumor cell behavior and the activities in the tumor microenvironment. Relevant bioinformatics analysis validation was also performed to ensure accuracy.

## 2. Materials and Methods

### 2.1. Screening of Differentially Expressed Genes

We downloaded the dataset of differentially expressed genes in lung adenocarcinoma from the TCGA database (https://tcga-data.nci.nih.gov/). From the gene list module of the immunology database and the analysis portal ImmPort database (ImmPort Private Data [nih.gov]), a total of 2483 immune genes was downloaded. We arranged the intersection of these data sets as a Wayne diagram and take the log2FC absolute value greater than 1 and *P* value less than 0.05 as the parameter to determine differentially expressed genes (DEGs).

### 2.2. Relationship between FGF11 in UALCAN Database and Clinical Data of Lung Adenocarcinoma

UALCAN (https://ualcan.path.uab.edu/) is a web-based program that analyzes transcriptome data from the Cancer Genome Atlas (TCGA). The association between FGF11 and clinicopathological characteristics of lung cancer was examined using UALCAN [17, 18].

### 2.3. The Relationship between FGF11 in KM-Plotter Database and Clinical Data of Lung Adenocarcinoma

The impact of clinical parameters and FGF11 expression on the clinical outcome of lung cancer was investigated using the Kaplan–Meier (KM)-plotter database (https://kmplot.com/analysis/index.php?p=service).

### 2.4. Nomogram Construction and Evaluation

We created a nomogram based on multivariate examination and expected survival rates of 1, 3, and 5 years. A nomogram showing clinical characteristics related to FGF11 and calibration plots were created using the rms package in R software. Calibration and discrimination are the most used methods for evaluating the performance of the models. In this study, the calibration curve was evaluated by mapping the nomogram prediction probability to the observed ratio with the 45° line representing the best prediction value. The consistency index (*C*-index) was used to determine the discrimination of nomographs, which was calculated using the bootstrap method with 1000 resamplings. In addition, the prediction accuracy of the nomogram and individual prognostic factors was compared using the *C*-index.

### 2.5. Analysis of FGF11-Interacting Genes and Proteins

The GeneMANIA database (https://www.genemania.org) was used to build the FGF11 interaction network. The protein-protein interaction (PPI) network of FGF11 was constructed using the STRING online database (https://cn.string-db.org).

### 2.6. Gene Ontology (GO) and Kyoto Encyclopedia of Genes and Genomes (KEGGs) Pathway Enrichment Analysis and Gene Set Enrichment Analysis (GSEA)

The biological function of FGF11 in lung cancer was investigated by GO and KEGG analyses. FGF11-related biological procedures (BPs), cellular mechanisms (CCs), and molecular activities were identified using GO analysis. The underlying mechanism of FGF11 expression was investigated using GSEA. GO, KEGG, and GSEA analyses were performed using the R package cluster profiler.

### 2.7. Correlation between FGF11 and Immune Cells in the TIMER Database

The Tumor Immune Estimation Resource (TIMER) database (https://cistrome.shinyapps.io/timer/) is an interactive portal that can comprehensively analyze the infiltration levels of different immune cells. In this study, FGF11 expression in various types of cancer was evaluated through the “diff exp” module. The correlation between FGF11 and immune cell infiltration in lung adenocarcinoma was analyzed using TIMER. The “gene” module was used with the TCGA database to study the relationship between FGF11 expression and the immune cell infiltration level (B cells, CD8+ T cells, CD4+ T cells, and others). TIMER was also used to evaluate the relationship between FGF11 expression and different gene marker sets of immune cells by using the “correlation” module. The correlation between FGF11 expression and immune infiltration was investigated using partial Spearman correlation and statistical significance related to tumor purity.

### 2.8. Evaluating Immune Cell Infiltration Using the CIBERSORT Algorithm

CIBERSORT (https://cibersort.stanford.edu/) is a computing resource for characterizing immune cell composition that is based on a validated leukocyte gene signature matrix, containing 547 genes and 22 human immune cell subsets. Our analysis measured the proportion of tumor infiltrating immune cells in lung adenocarcinoma by CIBERSORT and examined the correlation between FGF11 expression and immune cell subsets. *P* < 0.05 was used as the criterion for selecting lymphocytes that may be affected by FGF11 expression.

### 2.9. Statistical Analysis

The Kaplan–Meier diagram is presented with the hazard ratio and *p* values in the log-rank tests. The significance of continuous parameters expressed as the mean ± standard deviation was determined using Student's *t*-test. R(3.6.3) software (https://www.r.project.org/). The Wilcoxon test was used to compare the expression levels of FGF11 in normal and tumor tissues, while the Kruskal–Wallis one-way analysis of variance was used to evaluate the relationship between FGF11 expression and the clinical stage of the patients. The correlation between gene expression levels was evaluated using Spearman's correlation, and statistical significance was set at *P* < 0.05.

## 3. Results

### 3.1. Increased FGF11 Expression in Lung Adenocarcinoma

**Figure 1:**
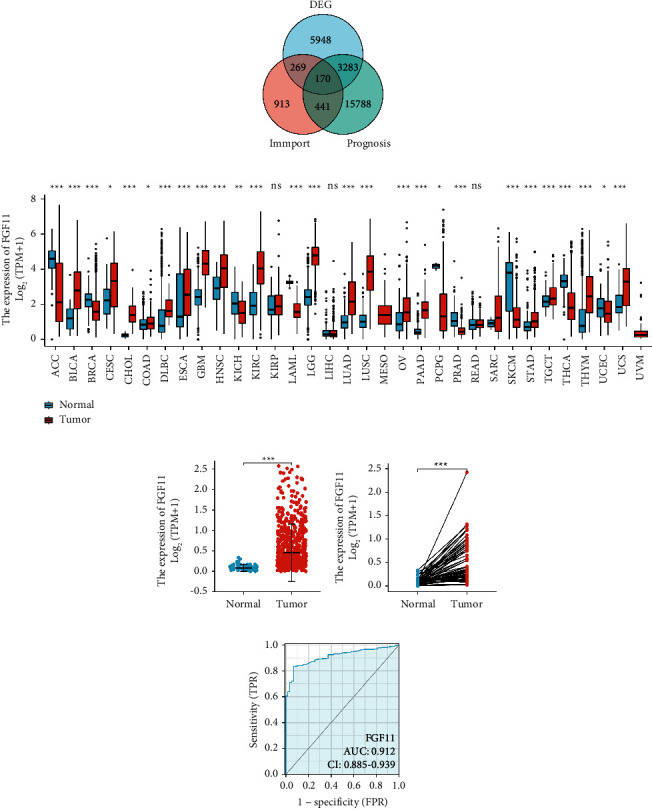
The expression of FGF11 elevated in lung adenocarcinoma. (a) Venn diagram showing the number of overlapped genes among indicated datasets. (b) FGF11 expression in the normal and tumor tissues of multiple cancers from TCGA database. (c) TCGA database search between unpaired lung adenocarcinoma (*n* = 535) and paracancerous tissues (*n* = 59). FGF11 is substantially expressed across tumors. (d) In the TCGA database, FGF11 is strongly expressed in matched bladder cancer tissues (*n* = 59) and paracancerous tissues (*n* = 59). (e) The ROC curve indicates FGF11 has a high predictive power to distinguish between tumor and normal tissues (^*∗*^*P* < 0.05, ^*∗∗*^*P* < 0.01, ^*∗∗∗*^*P* < 0.001).

First, we obtained data from TCGA database and ImmPort database and analyzed by Venn diagram, and we found that FGF11 is one of 170 differentially expressed genes, which is related to the prognosis of lung adenocarcinoma (Figure 1(a)). FGF11 was found to be substantially expressed in most tumor tissues after pan-cancer investigation (Figure 1(b)). The expression of FGF11 was also upregulated in lung adenocarcinoma tumor tissues according to the TCGA database (Figures 1(c) and 1(d)). The area under the curve was 0.912 in the receiver operating characteristic (ROC) curve, indicating that the high expression of FGF11 can further predict the poor prognosis of lung adenocarcinoma patients (Figure 1(e)).

### 3.2. The Relationship between FGF11 Expression and Clinical Data of Patients with Lung Adenocarcinoma

**Figure 2:**
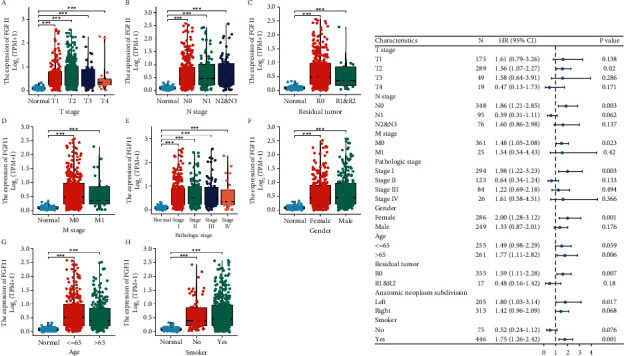
FGF11 expression in different patient groups evaluated based on clinical parameters using the UALCAN database. (a) FGF11 expression in patients with different TNM staging, pathological progression, age, and sex. (b) Forest map of FGF11 expression based on the clinical data in UALCAN database.

By analyzing the clinical data of bladder cancer in the UALCAN database, we found that the expression of FGF11 is different in patients with different TNM stages, different pathological stages, different ages, and different genders, but the expression in tumor tissues is higher than that in normal tissue. Meanwhile, the high expression of FEF11 is also consistent with the epidemiology that lung adenocarcinoma occurs more frequently in women and patients with a history of smoking (Figure 2(a)). Based on these clinical data, a forest map is drawn, and in most of the groups, FGF11 played the role of “risk factor,” which was consistent with the abovementioned results (Figure 2(b)).

### 3.3. Correlation between FGF11 Expression and Prognosis of Lung Adenocarcinoma Using the KM-Plotter Database

**Figure 3:**
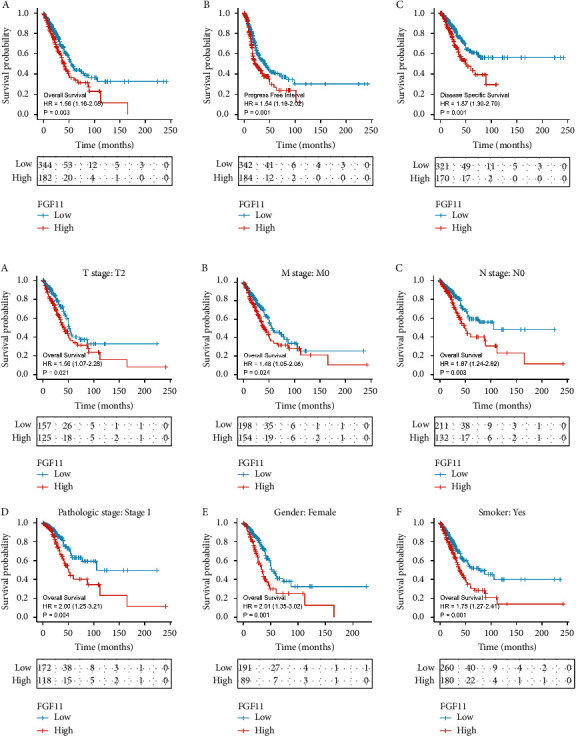
Expression of FGF11 and prognosis of patients with lung adenocarcinoma. (a) The relationship between FGF11 expression and LUAD patients' OS, PFS, and DSS. (b) The relationship between FGF11 and T2, M0, N0, stage I LUAD, smoking history, and prognosis in female patients with lung adenocarcinoma.

Using the lung cancer information within the KM-plotter database, we examined the prognosis of patients with varying FGF11 levels. Patients with lung adenocarcinoma with high FGF11 expression correlated with a shorter OS, PFS, and DSS than those with low FGF11 expression (Figure 3(a)). Further stratified patient analysis revealed that the patients with a lower FGF11 expression had a better prognosis than female patients, those with TMN staging of T2, M0, N0, stage I LUAD, and patients with a smoking history (Figure 3(b)).

### 3.4. Nomogram Construction

**Figure 4:**
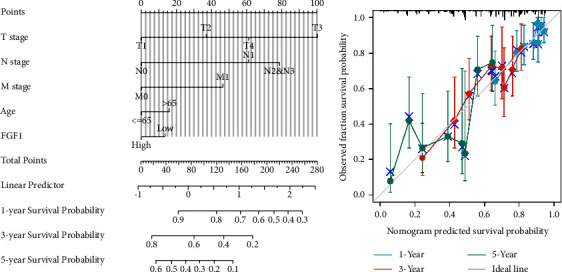
Nomograph and calibration chart. (a) Nomogram for predicting OS probability. (b) Calibration plot of nomogram predicting OS probability regarding patients with lung adenocarcinoma.

We created a nomogram based on multivariate analysis predicting the expected survival in patients with LUAD at 1, 3, and 5 years. This nomogram had a C-index of 0.679 (0.653–0.704). (Figure 4(a)). The bias correction line in the calibration plot is close to the ideal curve (45° line), indicating that the anticipated predicted values should be consistent with the real-world data (Figure 4(b)). These data indicate that the prediction model has a certain prediction accuracy.

### 3.5. Identification of FGF11-Interacting Genes and Proteins

**Figure 5:**
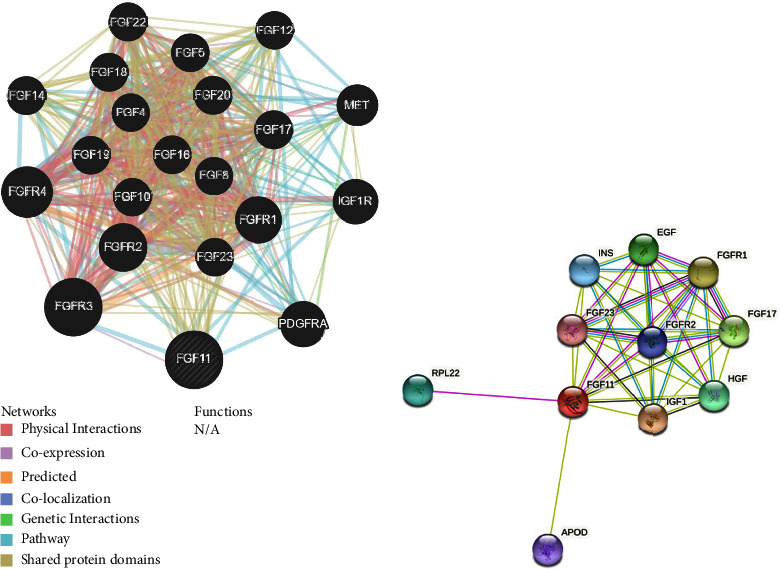
(a) FGF11 gene-gene interaction network was constructed using GeneMania. (b) PPI network of FGF11 was generated using the STRING database.

A gene-gene interaction network of FGF11 and altered adjacent genes was created by GeneMania (Figure 5(a)). The STRING database was used to create a protein-protein interaction (PPI) network for FGF11 (Figure 5(b)).

### 3.6. GO and KEGG Analyses of the FGF11 Pathway and Its Coexpressed Genes in Lung Adenocarcinoma Using the TCGA

**Figure 6:**
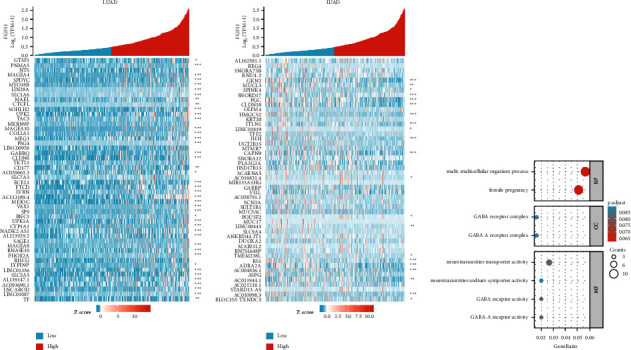
GO and KEGG enrichment analyses of FGF11. (a) Heatmap of the top 50 genes positively linked with FGF11 in lung adenocarcinoma. (b) Heatmap of the top 50 genes negatively correlated with FGF11 in lung adenocarcinoma. (c) Biological procedures (BP), cellular mechanisms (CC), and MF in lung adenocarcinoma.

Genes that were positively or negatively linked with FGF11 coexpression were identified using data from the TCGA database. We found the top 50 genes in lung adenocarcinoma that are positively and negatively correlated with FGF11 levels (Figures 6(a) and 6(b)). To uncover FGF11-related pathways and biological activities, we analyzed 600 FGF11-related genes using KEGG and GO enrichment analyses (Figure 6(c)).

### 3.7. Correlation between FGF11 Expression and Immune Cell Infiltration in the TIMER Database

**Figure 7:**
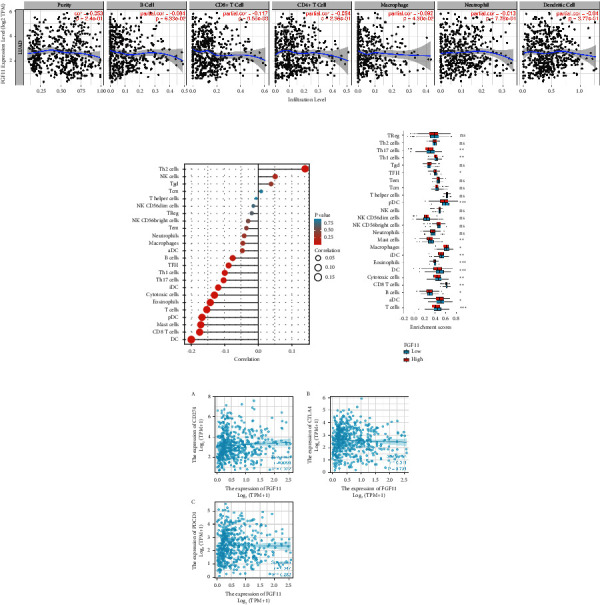
The expression of FGF11 is related to the infiltration of immune cells. (a) In the TIMER database, FGF11 is negatively correlated with infiltrating immune cells. (b) The expression of FGF11 in lung adenocarcinoma was significantly correlated with the infiltration of immune cells. (c) The expression of FGF11 and various immune cell subgroups in lung adenocarcinoma. (d) The scatter plot showed that FGF11, CTLA-4, and PDCD1 were the lack of expression in lung adenocarcinoma.

We used the TIMER database to analyze the association between FGF11 expression, tumor purity, and immune cell infiltration level in lung adenocarcinoma. We found an inverse relationship between FGF11 expression and six types of immune cells: B cells, CD4+ T cells, CD8+ T cells, neutrophils, macrophages, and dendritic cells (Figure 7(a)). We examined the relationship between FGF11 and selected immune cells to determine any effect on the tumor microenvironment (TME). FGF11 was shown to be negatively linked to dendritic cells, macrophages, neutrophils, T cells, Th1 cells, and other infiltrating cells but favorably associated with Th2 cells, NK cells, *γδ* T cells, and TCM infiltrating cells (Figures 7(b) and 7(c)). Additionally, P2RY14 expression correlated with the immunological checkpoint-related molecule CD274 (*P* < 0.05), but there was no significant correlation between CTLA-4 and PDCD1 expression (Figure 7(d)). Based on these results, we hypothesized that the FGF11 expression is linked to immune cell infiltration. These results suggest that in lung adenocarcinoma, FGF11 may play a key role in the immune escape of tumor cells, and these data indicate future directions for research.

### 3.8. Correlation between FGF11 Expression and Drug Target Molecules in the TCGA Database

**Figure 8:**
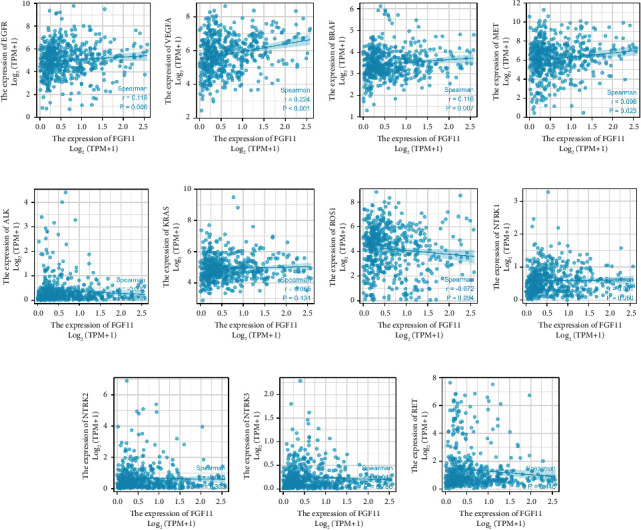
Correlation between FGF11 and molecules in the TCGA database that are currently used as therapy targets.

There are targeted therapies available for patients with advanced lung adenocarcinoma, and the most recent National Comprehensive Cancer Network (NCCN) lung cancer recommendations advocate for further identification of relevant drug targets, including mutant EGFR (19DEL, L858R). Osimertinib is the first-line therapy option for patients with cancer, followed by erlotinib, afatinib, gefitinib, dacomitinib, erlotinib + ramucirumab, and erlotinib plus bevacizumab. The ALK mutation is known as the “diamond mutation,” and treatments for ALK rearrangement-positive nonsmall cell lung cancer recommend alectinib, brigatinib, or lorlatinib as first-line therapy, with ceritinib or ceritinib as secondary alternatives. Thus, we conducted a correlation study between FGF11 and EGFR (19DEL, L858R), EGFR (Exon 20ins), KRAS (G12C), ALK, ROS1, BRAF, NTRK1/2/3, MET, and RET, which are all recommended by the NCCN guidelines for the diagnosis of nonsmall cell lung cancer. FGF11 was significantly associated with EGFR, VEGFA, BRAF, and MET (*P* < 0.05). However, there was no significant difference in the association between FGF11 and ALK, KRAS, ROS1, NTRK1, NTRK2, NTRK3, or RET (Figure 8).

### 3.9. Correlation between FGF11 Expression and Immune Cell Markers

We used the TIMER database to further evaluate the interaction between FGF11 and drug responses. We discovered a link between FGF11 expression and immune cell markers in lung adenocarcinoma. B cells, T cells, CD8+ T cells, monocytes, tumor-associated macrophages (TAMs), M1 macrophages, M2 macrophages, neutrophils, NK cells, and dendritic cells were used because we previously used them to discover immune-related genes in Table 1. The development of immune cell penetration resistance in clinical surgeries remains prejudiced through tumor purity. FGF11 expression remained substantially related to the greatest immunological indicators within distinct kinds of immune cells within lung adenocarcinoma after controlling for tumor purity (Table 1).

We also examined the relationship between the FGF11 expression and other types of T cells, such as Th1, Th1-like, and Th2. After controlling for tumor purity, we discovered that FGF11 expression levels were strongly linked to 12 T cell markers in lung adenocarcinomas using the TIMER database (Table 2).

T cell depleting therapies may concern patients with possibilities of chronic infections and future malignancies. T lymphocytes are abundant in patients with tumors, even though most of them are functionally exhausted [19, 20]. FGF11 has been shown to be negatively linked to the infiltration of dendritic cells, macrophages, neutrophils, T cells, Th1 cells, and immune cell genes. FGF11 expression was not correlated with functional T cell differentiation genes, such as Th1, Th1-like, Treg, and resting Tregs. Therefore, we hypothesize that FGF11 in the lung adenocarcinoma microenvironment increases tumor immune escape by increasing T cell depletion and exhaustion, contributing to the poor prognosis in patients with lung adenocarcinoma.

### 3.10. Correlation between FGF11 Expression and Regulators of T Cell Exhaustion

**Figure 9:**
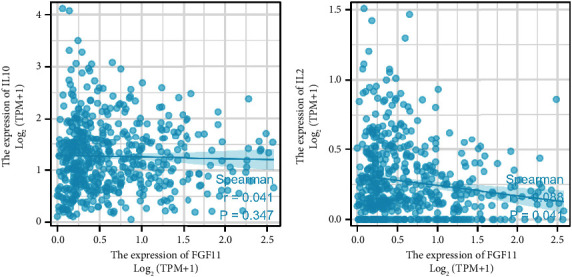
Correlation of FGF11 with regulators of T cell exhaustion. (^*∗*^*P* < 0.05).

We examined the relationship between FGF11 and T cell exhaustion regulators. FGF11 was found to have a positive correlation with IL-10 and a negative correlation with IL-2 (Figure 9) IL-10 is a STAT3-induced cytokine that promotes T cell depletion. Blocking IL-10 can prevent and/or reverse T cell depletion. Conversely, IL-2 is a critical cytokine that promotes T cell survival and activity, improves infection and tumor immune responses, and counteracts T cell depletion. This information supports our hypothesis.

## 4. Discussion

Many factors, including local invasion, distant metastasis, and treatment resistance, lead to poor outcomes in patients with lung adenocarcinoma [21]. In the United States, 228,000 individuals were diagnosed with lung cancer in 2019 and approximately 160,000 patients died [22]. Lung cancer has a high rate of morbidity, mortality, and poor prognosis. Of all nonsmall cell lung cancers (NSCLCs), lung adenocarcinoma is the most diagnosed subtype and entails a poor prognosis [23, 24]. As a result, modern lung adenocarcinoma research faces the challenge of identifying how LUAD develops, how it invades, and the process for distant metastasis. Targeted therapy, immunosuppressive therapy, and other treatment methods are now widely used in clinical practice, owing to many breakthroughs and advances in bioinformatics, molecular biology, immunology, and other fields, but these breakthroughs have not significantly decreased the mortality of patients with LUAD [25, 26]. Therefore, understanding the mechanisms of lung cancer incidence and progression is critical to uncovering significant biomarkers and identifying novel treatment options.

The development of sequencing and omics technologies nowadays has provided more opportunities to further understand the mechanism of lung adenocarcinoma and explore diagnostic and therapeutic targets [27]. We analyzed the TCGA database and other bioinformatics sources to determine whether FGF11 is associated with lung adenocarcinoma initiation and progression. We discovered that FGF11 expression is higher in lung adenocarcinoma tissues than in normal tissues. We also found that that patients with lung adenocarcinomas with a high FGF11 expression experienced lower OS, PFS, and DSS compared to those of patients with a low FGF11 expression. We used multivariate analysis of these data to build an accurate prediction model to assess the 1-, 3-, and 5-year survival likelihood of those patients with high levels of FGF11. Using the TIMER database, we discovered that higher FGF11 expression was correlated with decreased tumor infiltration of immune cells. These cells included B cells, CD4+ T cells, CD8+ T cells, neutrophils, macrophages, and dendritic cells. Additionally, we found that the higher expression of FGF11 is correlated with higher levels of the immune checkpoint-related molecule CD274. Thus, by decreasing immune cell infiltration and restricting the immune anticancer activity, FGF11 may play a role in increasing tumor cell immune escape in the tumor microenvironment of LUAD. We then examined the relationship between FGF11 and targets for drug therapy and discovered that FGF11 levels are linked to EGFR, VEGFA, BRAF, and MET levels in LUAD, of which LUAD is the most predominant subtype. According to these findings, FGF11 leads to a lower immune cell infiltration and can also alter medication selection and their effectiveness in LUAD.

Our data indicate the FGF11 function in immune aspect. Multiple regimens are now employed in clinical practice to enhance the prognosis of patients with progressive lung cancer, and recent clinical trials have used immune-focused therapies in their treatment plans. These regimens include immune therapy combined with chemotherapy, targeted drugs, and other immunotherapies. Potential future therapies are noted in the clinical studies, such as the KEYNOTE-799 clinical trial. The anti-PD-1 antibody pembrolizumab was used in combination with chemoradiation treatment for unresectable, locally advanced, stage III NSCLC [28]. The KEYLYNK-012 trial is investigating a treatment strategy for unresectable stage III NSCLC using PARP inhibitors combined with pembrolizumab and chemoradiotherapy [29]. The updated data from the CheckMate 9LA study show that when compared to four cycles of chemotherapy alone, dual immunotherapy (nivolumab and ipilimumab) combined with short-course chemotherapy treatment-naïve patients with progressive NSCLC increased patient OS, overcoming the low response rate of a single immunotherapy while maximizing the long-tail effect of immunotherapy [30]. Furthermore, the CheckMate 816 study [31] corroborated the effectiveness of neoadjuvant combination immunotherapy (nivolumab) with chemotherapy in resectable NSCLC, indicating that another treatment may be possible for the patients with NSCLC.

The therapeutic and scientific relevance of our research is reflected in the immunity-focused results. We examined the relationship between FGF11 and immunological markers and found that FGF11 inversely correlated with all genes that characterize immune cells, including functional T cell characterization genes such as Th1, Th1-like, Treg, and resting Tregs. This prompted us to investigate whether FGF11 in the immunological milieu of lung adenocarcinoma increases tumor cell immune escape by boosting T cell exhaustion, thereby contributing to the poor prognosis of lung adenocarcinoma.

T cell exhaustion indicates decreased T cell function in patients with prevalent chronic illnesses or malignancies. Exhausted T cells eventually lose the effector activity and memory T cell properties because of extended exposure to antigens or chronic inflammation. However, this exhaustion may be partially restored by blocking inhibitory mechanisms, such as PD-1 and IL-10. The persistent exposure of T cells to antigens is a characteristic element of chronic infection or malignancy, and both high antigen load and extended antigen exposure contribute to more severe T cell exhaustion [32, 33]. The STAT3-induced cytokine, IL-10, produces T cell exhaustion and reduces T cell activation. T cell exhaustion was prevented and/or reversed when IL-10 was blocked. Dendritic cells, B cells, monocytes, CD8+ T cells, and nonregulatory CD4+ T cells are among the immune cell types that may release IL-10 [34]. T cells may be directly affected by IL-10 via STAT3, indirectly affected by APC modulation, or both. Neutralizing this IL-10 effect with antibodies combined with immunotherapy may improve CD8+ and CD4+ T cell effector responses [35]. IL-2 is a critical cytokine required for T cell survival and activation as well as for increasing infection and tumor immune responses. It belongs to a family of cytokines that counteract T cell depletion. IL-2 levels are increased after the use of a microRNA targeting the mRNA of T cell inhibitory receptors PD-1, TIM-3, BTLA, and Foxp1, indicating its positive role on anticancer immune responses [36, 37]. FGF11 was shown to have a positive correlation with IL-10 and a negative correlation with IL-2, indicating how it negatively modulates immune reactions in the TME. These findings support our hypothesis that FGF11 increases T cell exhaustion.

## 5. Conclusion

This study adds to the body of data supporting FGF11's role in LUAD formation and as a potential biomarker of LUAD. FGF11 may increase tumor cell immune escape by increasing T cell exhaustion in the LUAD tumor microenvironment, contributing to the poor prognosis for patients with LUAD. These results suggest that FGF11 is a possible target for future lung adenocarcinoma anticancer treatments.

## Figures and Tables

**Table 1:** Correlation analysis between FGF11 and immune-cell related gene markers using the TIMER data.

Descriptions	Gene markers	LUAD			
		None		Purity	
		Cor	*P*	Cor	*P*
B cell	CD19	0.002	^*∗∗∗*^	−0.492	^*∗∗∗*^
	CD79A	0.036	^*∗∗∗*^	−0.457	^*∗∗∗*^

T cell (general)	CD3D	−0.086	^*∗∗∗*^	−0.517	^*∗∗∗*^
	CD3E	−0.093	^*∗∗∗*^	−0.536	^*∗∗∗*^
	CD2	−0.087	^*∗∗∗*^	−0.522	^*∗∗∗*^

CD8+ T cell	CD8A	−0.074	^*∗∗∗*^	−0.437	^*∗∗∗*^
	CD8B	−0.057	^*∗∗∗*^	−0.348	^*∗∗∗*^

Monocyte	CD86	−0.025	^*∗∗∗*^	−0.45	^*∗∗∗*^
	CSF1R	−0.008	^*∗∗∗*^	−0.396	^*∗∗∗*^

TAM	CCL2	0.079	^*∗∗∗*^	−0.334	^*∗∗∗*^
	CD68	−0.011	^*∗∗∗*^	−0.659	^*∗∗∗*^
	IL10	−0.03	^*∗∗∗*^	−0.42	^*∗∗∗*^

M1	IRF5	0.014	^*∗∗∗*^	−0.337	^*∗∗∗*^
	PTGS2	0.165	^*∗∗∗*^	−0.019	^*∗∗∗*^
	NOS2	0.066	^*∗∗∗*^	−0.23	^*∗∗∗*^

M2	CD163	−0.002	^*∗∗∗*^	−0.385	^*∗∗∗*^
	VSIG4	−0.063	^*∗∗∗*^	−0.33	^*∗∗∗*^
	MS4A4A	−0.113	^*∗∗∗*^	−0.409	^*∗∗∗*^

Neutrophils	CEACAM8	−0.165	^*∗∗∗*^	−0.066	^*∗∗∗*^
	ITGAM	−0.009	^*∗∗∗*^	−0.363	^*∗∗∗*^
	CCR7	−0.127	^*∗∗∗*^	−0.522	^*∗∗∗*^

Natural killer cell	KIR2DL1	−0.083	^*∗∗∗*^	−0.153	^*∗∗∗*^

Dendritic cell	HLA-DPB1	−0.161	^*∗∗∗*^	−0.388	^*∗∗∗*^

(^*∗∗∗*^: [*P* ≤ 0.001]).

**Table 2:** Correlation analysis between FGF11 and T cell gene markers using the TIMER data.

Descriptions	Gene markers	LUAD			
		None		Purity	
		Cor	*P*	Cor	*P*
Th1	TBX21	−0.072	^*∗∗∗*^	−0.452	^*∗∗∗*^
	STAT4	−0.033	^*∗∗∗*^	−0.456	^*∗∗∗*^
	STAT1	0.113	^*∗∗∗*^	−0.329	^*∗∗∗*^
	TNF	0.049	^*∗∗∗*^	−0.398	^*∗∗∗*^
	IFNG	0.035	^*∗∗∗*^	−0.349	^*∗∗∗*^

Th1-like	CXCR3	−0.065	^*∗∗∗*^	−0.432	^*∗∗∗*^
	BHLHE40	0.051	^*∗∗∗*^	−0.106	^*∗∗∗*^
	CD4	−0.084	^*∗∗∗*^	−0.478	^*∗∗∗*^

Th2	STAT6	−0.151	^*∗∗∗*^	0.026	^*∗∗∗*^
	STAT5A	−0.071	^*∗∗∗*^	−0.414	^*∗∗∗*^

Treg	FOXP3	−0.011	^*∗∗∗*^	−0.476	^*∗∗∗*^

Resting treg	IL2RA	−0.003	^*∗∗∗*^	−0.388	^*∗∗∗*^

(^*∗∗∗*^: [*P* ≤ 0.001]).

## Data Availability

The datasets used or analyzed to support the findings of this study are available from the corresponding author on reasonable request.
